# Ageing-related structural and cellular alterations in the mouse muscle-tendon junction

**DOI:** 10.1007/s10522-026-10428-x

**Published:** 2026-04-04

**Authors:** Chavaunne T. Thorpe, Nodoka Iwasaki

**Affiliations:** Comparative Biomedical Sciences, Royal Veterinary College, Royal College Street, London, UK

**Keywords:** Muscle tendon junction, Ageing, µCT, Immunolabelling, Endothelial cells, Senescence

## Abstract

The muscle-tendon junction (MTJ) is a specialised interface between muscle and tendon and transmits muscle-generated force to the tendon. The MTJ is particularly vulnerable to injuries compared to muscle and tendon and becomes more injury prone with age. Despite its clinical importance, the mechanisms driving MTJ ageing and age-related functional deterioration remain poorly understood. In this study, young (3-month-old) and old (23-month-old) male mice were used to provide the first comprehensive three-dimensional characterisation of age-related structural and cellular changes at the mouse Achilles MTJ. This was achieved using the high-resolution imaging techniques, micro-computed tomography (µCT) and confocal microscopy. µCT analysis revealed a 27% reduction in muscle fibre diameter with age, accompanied by a trend toward increased MTJ surface area and a 19% reduction in pennation angle, which may indicate diminished force generation capacity. Confocal imaging showed a 49% reduction in endothelial cell volume (VWF-labelled) in the old mouse muscle-tendon unit, suggesting a loss of vascularity. In situ hybridisation demonstrated increased expression of senescence markers p16 and p21 in endothelial and MTJ-specific cells, with MTJ-specific cells showing the greatest accumulation of p16 and p21 (270% and 310% increases, respectively) with age, and immunofluorescence also showed increased expression of p21. These findings suggest that vascular and MTJ-specific cells are particularly susceptible to ageing and may collectively contribute to the age-related functional decline of the MTJ. Understanding these mechanisms may help to develop targeted therapeutic strategies to preserve or restore MTJ integrity and function in ageing populations.

**Supplementary Information:**

The online version contains supplementary material available at 10.1007/s10522-026-10428-x.

## Introduction

The muscle-tendon junction (MTJ), also known as the myotendinous junction, is a specialised interface between muscle and tendon, and transmits the force generated by the muscle to its connecting tendon (Tidball 1983). The MTJ is commonly associated with muscle strains and tears (Huijing 1999) and is particularly vulnerable to tensile failures compared to the neighbouring muscle and tendon (Iwasaki et al. 2024a, 2025). Injuries and failure at the MTJ increase the morbidity of patients and affect their quality of life (Clarkson and Hubal 2002; Zhao et al. 2018).

MTJ injuries are common, with 28% of injuries in the muscle-tendon-bone unit and 52% of acute hamstring injuries occurring at the MTJ (Garrett and Faherty 2017; Vila Pouca et al. 2021; Grange et al. 2023). In the non-athletic population, injuries at the gastrocnemius-Achilles myoaponeurotic junction occur at an older age (48.7 ± 8.1 years) (Pedret et al. 2020) compared to tendon injuries (~ 40 years) (Ho et al. 2017; Čretnik and Košir 2023), suggesting increased vulnerability with ageing. Moreover, 2D structural studies in ageing rodents have revealed that the length of the MTJ region approximately doubles with age (Nielsen et al. 2018), indicating that the MTJ undergoes degenerative changes with age and may contribute to an increased risk of injury. However, the specific effects of ageing on MTJ structure and function remain poorly understood (McCarthy and Hannafin 2014). This gap in knowledge limits our understanding of the mechanisms underlying age-related MTJ deterioration and hinders advances in the discovery of targeted therapeutic strategies.

The extracellular matrix (ECM) of the MTJ contains MTJ-specific adhesion proteins, including collagen type XXII (Col22), paxillin, and talin (Koch et al. 2004; Charvet et al. 2012). These proteins are critical for efficient force transmission between muscle and tendon and play key roles in maintaining MTJ structural and functional integrity (Bayrak and Yilgor Huri 2018). Col22 is one of the most well-characterised MTJ-specific proteins, which is expressed exclusively at the MTJ by both muscle cells and tenocytes (Koch et al. 2004; Petrany et al. 2020; Iwasaki et al. 2024b; Møbjerg et al. 2025), and has been reported to maintain vascular integrity by regulating vascular permeability (Ton et al. 2018). Ageing significantly affects the vasculature in the musculoskeletal system, and both aged muscle and tendon have been reported to exhibit reduced vascularisation and blood flow (Brewer 1979; Rudzki et al. 2008; Márquez-Arabia et al. 2017; Marqueti et al. 2018; Socha and Segal 2018; Fukada and Kajiya 2020; Iwasaki et al. 2026), which may contribute to the higher incidence of injury and impaired regenerative capacity observed with age. However, it remains to be established if the MTJ vasculature is similarly affected by ageing.

Cellular senescence, a state of permanent cell cycle arrest triggered by various stressors, plays a critical role in the ageing process, with the proportion of senescent cells increasing across multiple tissues with age (Saito et al. 2024). In muscle and tendon, senescence is linked to impaired regeneration, reduced differentiation capacity, and functional decline (Baker et al. 2011; Han et al. 2017; Englund et al. 2023). Senescence also affects vascular cells, where it disrupts angiogenesis and tissue repair (Bloom et al. 2023; Han and Kim 2023), and treating endothelial cell senescence may therefore enhance tissue regenerative capacity. Endothelial cell senescence has been demonstrated in several tissues, including brain, kidney and tendon (Han and Kim 2023; Zamboulis et al. 2024). While these findings indicate that MTJ-localised cells are likely to suffer from senescence, the precise effects of senescence on the ageing MTJ remain to be elucidated.

Obtaining healthy human MTJ is challenging, therefore, appropriate models are needed to study the effects of ageing in healthy MTJ. Mouse models are widely used in ageing research due to their low cost, short lifespan, and physiological and cellular similarities to humans including comparable musculoskeletal, immune, endocrine, and digestive systems (Vanhooren and Libert 2013). Indeed, mouse models are commonly used to study the MTJ in the context of injury, ageing and development (Nielsen et al. 2018; Yaseen et al. 2021; Iwasaki et al. 2024a, 2025). C57BL/6 mice are among the most widely used strains in ageing research (Nadon 2004) and were therefore selected for this study.

The aim of this study was to investigate age-related alterations in MTJ structure, vasculature and cellular senescence, testing the hypothesis that MTJ undergoes age-related structural and cellular alterations, with particular involvement of MTJ-specific cell populations and vascular cells. Understanding these structural and cellular alterations with age may provide insight into the mechanisms underlying MTJ degeneration and support the development of targeted therapeutic strategies to enhance MTJ repair and regeneration.

## Materials and methods

### Sample acquisition

Mouse hindlimbs were obtained as residual tissues from a separate study (a kind gift from Dr. Linterman, Babraham Institute). In that study, male C57BL/6 mice were used, comprising two age groups: young (3 months old, 29.2 ± 1.3 g; equivalent to 20–30 human years) and old (23 months old, 44.6 ± 3.2 g; equivalent to 56–69 human years) (Dutta and Sengupta 2016), with n = 6 per group. All mice underwent the same experimental procedure, receiving an intramuscular injection of 50 µg/mL lipid nanoparticle (LNP)-mRNA vaccine encoding the ancestral SARS-CoV-2 spike protein into the right biceps femoris muscle. Ten days post-injection, animals were euthanised and hindlimb tissues were collected for downstream use. All procedures were approved by the Babraham Institute Animal Welfare and Ethical Review Body and conducted in accordance with European Union and UK Home Office regulations (Home Office Licence P4D4AF812).

Achilles tendon and gastrocnemius muscle junctions (Achilles MTJs) were subsequently harvested from both hindlimbs (12 hindlimbs in total per age group) of the immunised mice 3 h after euthanasia at the Royal Veterinary College. The experiments were approved by the Royal Veterinary College Clinical Research Ethical Review Board (URN 2024–2336-A). Harvested MTJs were divided into three groups randomly; with MTJs from the same mouse being distributed across different groups. Samples were subsequently processed for µCT imaging, 3D immunolabeling, or 2D analysis (in situ hybridisation and 2D immunolabelling) (n = 4 per age group, Fig. S1).

### µCT imaging

Phosphotungstic acid (PTA) was used to enhance the contrast of the MTJ, adapting a protocol from a previous study (Iwasaki et al. 2024a). Young and old Achilles MTJs were immersed in an increasing ethanol concentration of 25, 50, and 70% ethanol for 90 min each, followed by 1% PTA (79,690, Sigma-Aldrich, Burlington, MA, USA) in 70% ethanol for 72 h (n = 4 per age group). Samples were then washed twice and immersed in tris-buffered saline (TBS) for 30 min prior to imaging. Samples were wrapped in clingfilm to avoid dehydration during imaging. A Skyscan 1172F (version 1.5, Skyscan, Kontich, Belgium) was used with an X-ray source at 50 kV tube voltage and 200 μA tube current with 2500 ms exposure time. The voxel size was 2 μm, and 180° scans were performed with 0.5 mm Aluminium filter, frame averaging at 2, and with a rotation step at 0.25°. Slice reconstruction was performed using NRecon (version 1.7.1.0). The reconstructed images were segmented to remove tendons and analysed using CTAn (version 1.17.7.1) to measure mean muscle fibre diameter. CTVox (version 3.3.0) was used to visualise the 3D reconstructed images. The images were also analysed using Avizo (Avizo 2021.1, ThermoFisher Scientific, MA, USA), and the images were cropped at 2000 × 2000 × 1000 voxels (32 mm^3^) with the MTJ in the region of interest. Pennation angle was measured manually in Avizo using a 2D slice image at each muscle-tendon sub-unit interface at the MTJ. The angle was defined as the angle between the longitudinal axis of the tendon sub-unit and the orientation of the adjacent muscle fascicles (Figure S2a). For each sample, all identifiable interfaces on both the medial and lateral sides of the MTJ were measured, and the mean of these values was used as the representative pennation angle. The same measurement approach (bilateral MTJ, all visible sub-units) was applied consistently across all samples. No fixed number of sub-units was imposed; instead, all discernible bundles within each image (approximately 3 bundles per sample) were included to avoid selection bias. Tendon sub-unit diameter was also measured manually in Avizo. The images were then segmented to separate muscle and tendon, and the MTJ surface area was measured using the volume fraction function in Avizo (Figure S2b). To account for inter-sample variability arising from differences in overall tissue size among mouse samples, MTJ surface area was normalised to the total tissue surface area.

### 3D immunolabelling

MTJs from young and old mice were fixed in 4% paraformaldehyde (PFA) for 4 h for 3D immunolabelling (n = 4 per age group), using a protocol adapted from a previous study (Marr et al. 2020). Permeabilization was performed using 50% (v/v) methanol:TBS, 80% (v/v) methanol:dH2O, and 100% methanol for 2 h, and 20% (v/v) dimethylsulphoxide (DMSO):methanol, 80% (v/v) methanol:dH2O, 50% (v/v) methanol:TBS for 30 min at 4 °C, respectively, with gentle shaking. The samples were stored in TBS overnight at 4 °C. Blocking was performed using blocking solution (0.2% Triton X-100, 6% donkey serum, 6% goat serum, 10% DMSO in TBS) for 72 h at 37 °C with gentle shaking. The samples were then incubated with primary antibodies diluted in blocking solution for 72 h at 37 °C with gentle shaking. The details of primary antibodies were as follows: Rabbit anti-von Willebrand factor (VWF, endothelial cell marker, 1:800, A0082, Dako, Ejby, Denmark) and rat anti-laminin alpha 2 (LAMA2, skeletal muscle marker, 1:1000, ab11576, Abcam, Cambridge, UK). The samples were then washed 5 times with 0.2% Tween-20 in TBS for 1 h each at room temperature. The samples were incubated with secondary antibody diluted in blocking solution. The details of the secondary antibodies were as follows: Goat anti-rabbit IgG AF594 (1:800, A-11012, ThermoFisher Scientific, MA, USA) and Goat anti-rat IgG AF488 (1:800, ab150157, Abcam, Cambridge, UK) for 24 h at 37 °C with gentle shaking, followed by 5 washes with 0.2% Tween-20 in TBS for 1 h at room temperature. Samples were then incubated in DAPI solution (5 µg/mL in TBS) at 4 °C overnight. Samples were dehydrated as described above with increasing concentrations of methanol. Two-step tissue clarification was performed by immersing samples in Visikol HISTO-1 (H1-30, Sigma-Aldrich, MA, USA) for 24 h, followed by immersion in HISTO-2 (H2-30, Sigma-Aldrich, MA, USA) for at least 48 h at room temperature with gentle shaking.

### Confocal imaging

The 3D immunolabelled samples were placed in a glass-bottom dish fitted with a polystyrene frame (220.220.042, IBL Baustoff + Labor GmbH, Austria) and a drop of Histo-2 was added to keep the sample hydrated. The samples were then imaged using a Leica TCS SP8 laser scanning confocal microscope (Leica Biosystems, Nussloch, Germany) with 10 × objective, 512 × 512 pixel resolution with 2.27 µm pixel size and 2.27 µm z axis steps. The pinhole size was set to 1 Airy unit, frame average was set to 1, and line average was set to 2 using lasers emitting light at 405 nm (blue channel), 488 nm (green channel), and 561 nm (red channel). The images were visualised using Leica LAS X software (version 3.5.5) within the 3D module and reconstructed and analysed using Avizo. The reconstructed volume of immunolabeled vasculature was measured using the volume fraction function in Avizo.

### In situ hybridisation

Mouse MTJs were snap frozen in hexane cooled on dry ice (n = 4 per age group), and then embedded using OCT (15,212,776, ThermoFisher Scientific, MA, USA). In situ hybridisation was performed using OCT-embedded MTJ sections and RNAscope Multiplex Fluorescent Reagent Kit v2 (323,110, Bio-Techne Ltd, MN, USA) following the manufacturer’s protocol for fresh frozen tissues. The sections were fixed with 4% PFA for 2 h at 4 °C and then dehydrated using an increasing ethanol concentration of 50, 70, and 100% ethanol for 5 min each at room temperature, followed by -20 °C overnight incubation in 100% ethanol. The samples were dried for 5 min and encircled with a hydrophobic barrier pen, then incubated with hydrogen peroxide for 10 min and rinsed in distilled water. Custom pretreatment (300,040, Bio-Techne Ltd, MN, USA) was added for 30 min at 40 °C, followed by two washes with distilled water. Probe solution was applied to the tissue sections for 2 h at 40 °C. The details of the probes were as follows: Col22A1 (590,911, C1, Bio-Techne Ltd, MN, USA), VWF (499,111-C3, Bio-Techne Ltd, MN, USA), p16 (411,011-C2, Bio-Techne Ltd, MN, USA) and p21 (408,551-C2, Bio-Techne Ltd, MN, USA). The samples were stored overnight at room temperature in 5 × saline-sodium citrate buffer (0.75 M sodium chloride, 75 mM trisodium citrate, pH 7.0). On the following day, signal amplification was performed according to the manufacturer’s instructions. All incubations were conducted at 40 °C, followed by two 2-min washes with RNAscope wash buffer. Amplifier incubations were carried out for 30 min for the first two amplifiers and 15 min for the third. Subsequently, slides were incubated with horseradish peroxidase (HRP) for 15 min, followed by a 30-min incubation with a tyramide dye fluorophore (OPAL 520, FP1487001KT, Akoya Biosciences, MA, USA) diluted 1:1500 in RNAscope TSA dilution buffer, and a 30-min incubation with HRP blocker. The HRP, fluorophore, and blocking steps were repeated using second and third tyramide dye fluorophores (OPAL 570 and OPAL 650, FP1488001KT and FP1496001KT, Akoya Biosciences, MA, USA). The sections were then incubated with DAPI for 30 s at room temperature, followed by mounting with ProLong™ Gold Antifade Mountant (P10144, ThermoFisher Scientific, MA, USA) and allowed to cure for 2–3 h before imaging using an Eclipse Ni-E upright microscope (Nikon Instruments Inc., Tokyo, Japan). Four images were obtained per sample, and they were analysed using ImageJ (National Institutes of Health, Austin, USA) by manually counting cells expressing the positive signals of target RNAs (Col22, VWF, p16 and p21). The percentages of p16 and p21 positive cells within Col22-positive MTJ-specific cell and VWF-positive endothelial cell populations were quantified and compared with their prevalence among all cells within the field of view.

### 2D immunolabelling

2D immunolabelling was performed using OCT-embedded MTJ sections (n = 4 per age group). Longitudinal MTJ cryosections (10 μm thickness) were fixed in ice-cold methanol/acetone solution (1:1) for immunolabelling. Non-specific binding of antibodies was blocked by incubating samples with 5% goat serum (ab7481, Abcam, Cambridge, UK) in TBS for 45 min at room temperature. The samples were incubated with primary antibodies in 5% goat serum for 2 h at room temperature. The details of primary antibodies were as follows: Guinea‐pig anti‐collagen XXII (1:100, monoclonal, a kind gift from Manuel Koch, University of Cologne, Germany) and rabbit anti-p16 (1:50, 80772S, Cell Signaling Technology, MA, USA). After washing twice with TBS, the sections were incubated with secondary antibodies (A-11012 and SA5-10,096, ThermoFisher Scientific) in 5% goat serum (1:400) for 1 h at room temperature. The sections were then incubated with DAPI (0.1 µg/mL) for 10 min at room temperature, followed by two washes in TBS. The sections were mounted with ProLong™ Gold Antifade Mountant (P10144, ThermoFisher Scientific, MA, USA) and allowed to cure for 2–3 h before imaging using an Eclipse Ni-E upright microscope (Nikon Instruments Inc., Tokyo, Japan). Four images were obtained per sample, and they were analysed using ImageJ by manually counting positively labelled cells.

### Statistical analysis

All data are expressed as the mean ± standard deviation (SD), and all experiments were conducted using 4 different animals from each age group. A D’Agostino and Pearson test was used to determine if the data followed a normal distribution. The Mann-Whitney test was performed to calculate the differences (p < 0.05) between different age groups and two-way ANOVA followed by Tukey’s multiple comparisons test was performed to calculate the differences (p < 0.05) between different age groups and cell populations using GraphPad Prism version 10.2.3 (La Jolla, CA, USA).

## Results

### µCT analysis demonstrated age-related structural changes in mouse MTJs

**Fig. 1:**
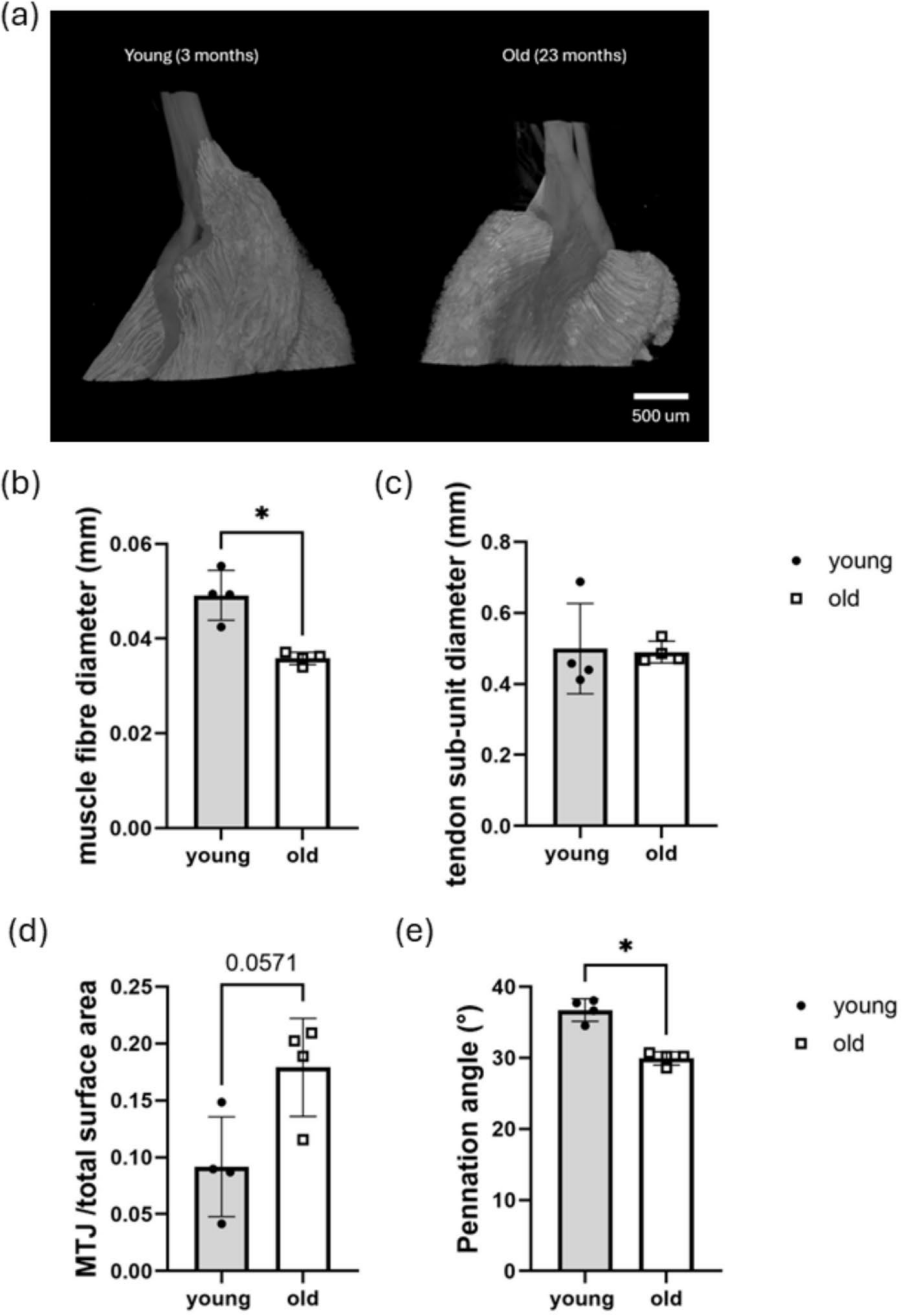
µCT image analysis shows significant decreases in muscle fibre diameter and pennation angle at the MTJ with age. **a** Representative 3D reconstructed µCT images of young and old MTJ in longitudinal view. Scale bar is 500 µm. Quantitative analysis of µCT images showing **b** muscle fibre diameter, **c** tendon sub-unit diameter, **d** MTJ surface area normalised to total surface area, and **e** pennation angle at the MTJ. Data are presented as mean ± SD from analysis of 4 young and 4 old mice. Mann-Whitney test was used to calculate the significance between young and old MTJs. *p < 0.05

Quantitative analysis revealed a 27% reduction in muscle fibre diameter with age in the MTJ region (Fig. 1b), whereas tendon sub-unit size was not significantly affected (Fig. 1c). The MTJ surface area, normalised to total surface area, showed a trend towards an age-related increase (p = 0.0571; Fig. 1d), suggesting enlargement of the MTJ with ageing. The pennation angle, an indicator of muscle force generation capacity, was significantly reduced by 19% in old mouse MTJs compared with those of young mice (Fig. 1e). The muscle and tendon volume at the MTJ, normalised to the whole MTJ volume, showed no significant difference with age (Figure S3).

### Three-dimensional immunolabelling demonstrated a significant reduction in VWF labelled endothelial cell volume with age

**Fig. 2:**
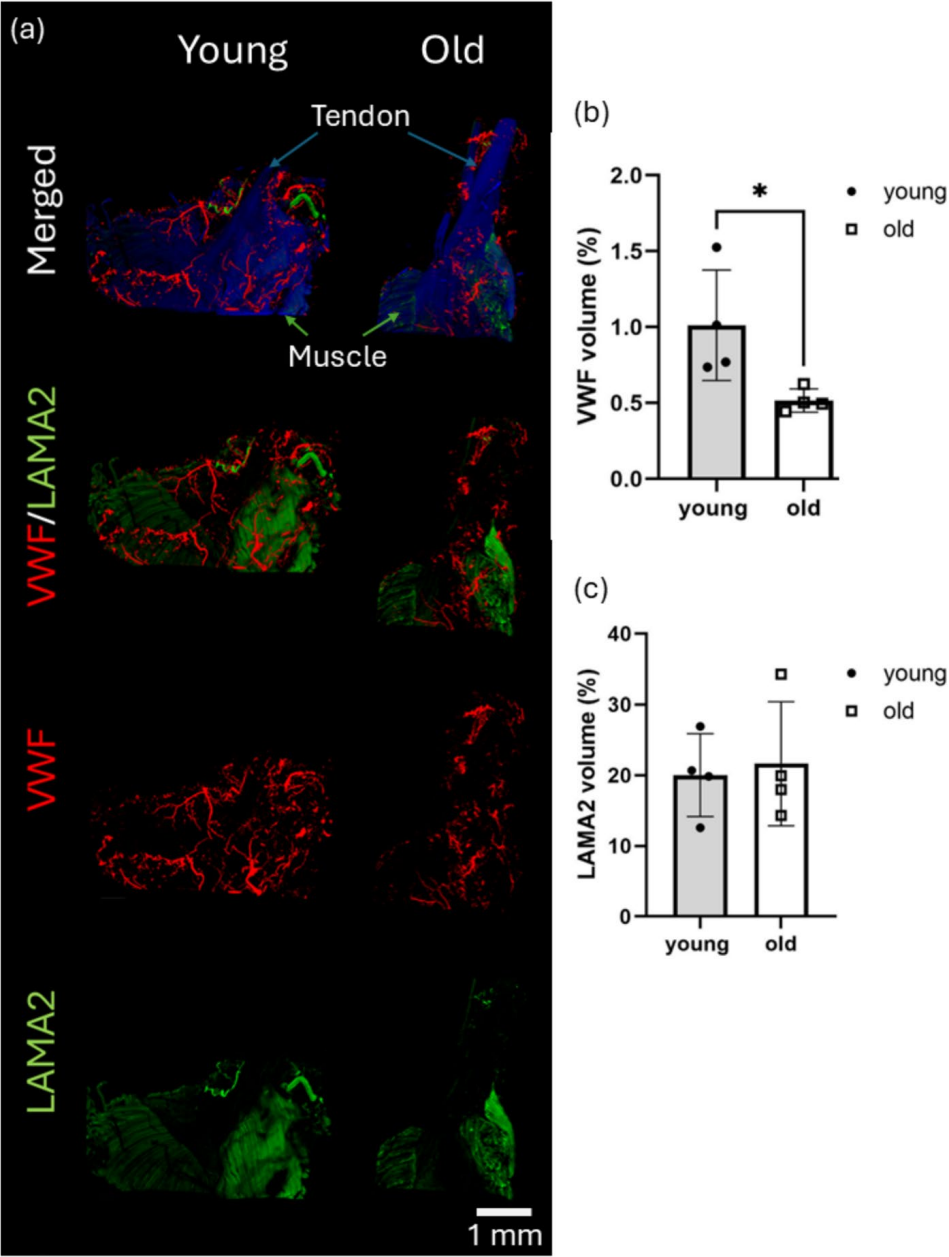
Immunolabeled 3D image analysis shows a significant decrease in muscle-tendon unit vascularity with age. **a** Representative reconstructed 3D immunolabelled images of young and old mouse MTJ with surrounding muscle and tendon (n = 4 per age group) with endothelial cell marker, VWF (red), muscle marker, LAMA2 antibodies (green), and DAPI (blue). Scale bar is 1 mm. Immunolabelled volume of **b** VWF (endothelial cell marker) and **c** LAMA2 (skeletal muscle marker) in young and old MTJ normalised using DAPI labelled volume. Data are presented as mean ± SD (n = 4). A Mann-Whitney test was used to calculate the significance between young and old MTJs. *p < 0.05

Whole-tissue immunolabelling was performed to visualise endothelial and muscle cell populations within the muscle-tendon unit including the MTJ. VWF was used to label endothelial cells and LAMA2 was used to label muscle cells, and DAPI staining was applied to identify the overall tissue volume. The images showed that the vasculature exists not only in the muscle, as indicated by LAMA2 labelling, but also within the tendon and across their interfaces, demonstrating the presence of vasculature throughout the MTJ region (Fig. 2a).

Quantitative analysis of VWF- and LAMA2-labelled volumes revealed a significant reduction of 49% in VWF-labelled volume in old mice (Fig. 2b), whereas LAMA2-labelled volume showed no significant change with age (Fig. 2c). This finding indicated an age-associated decline in vascularity within the muscle-tendon unit, without a corresponding reduction in muscle volume.

### Collagen type 22 positive MTJ-specific cells and VWF-positive endothelial cells exhibit higher expression of senescence markers at the MTJ region

**Fig. 3:**
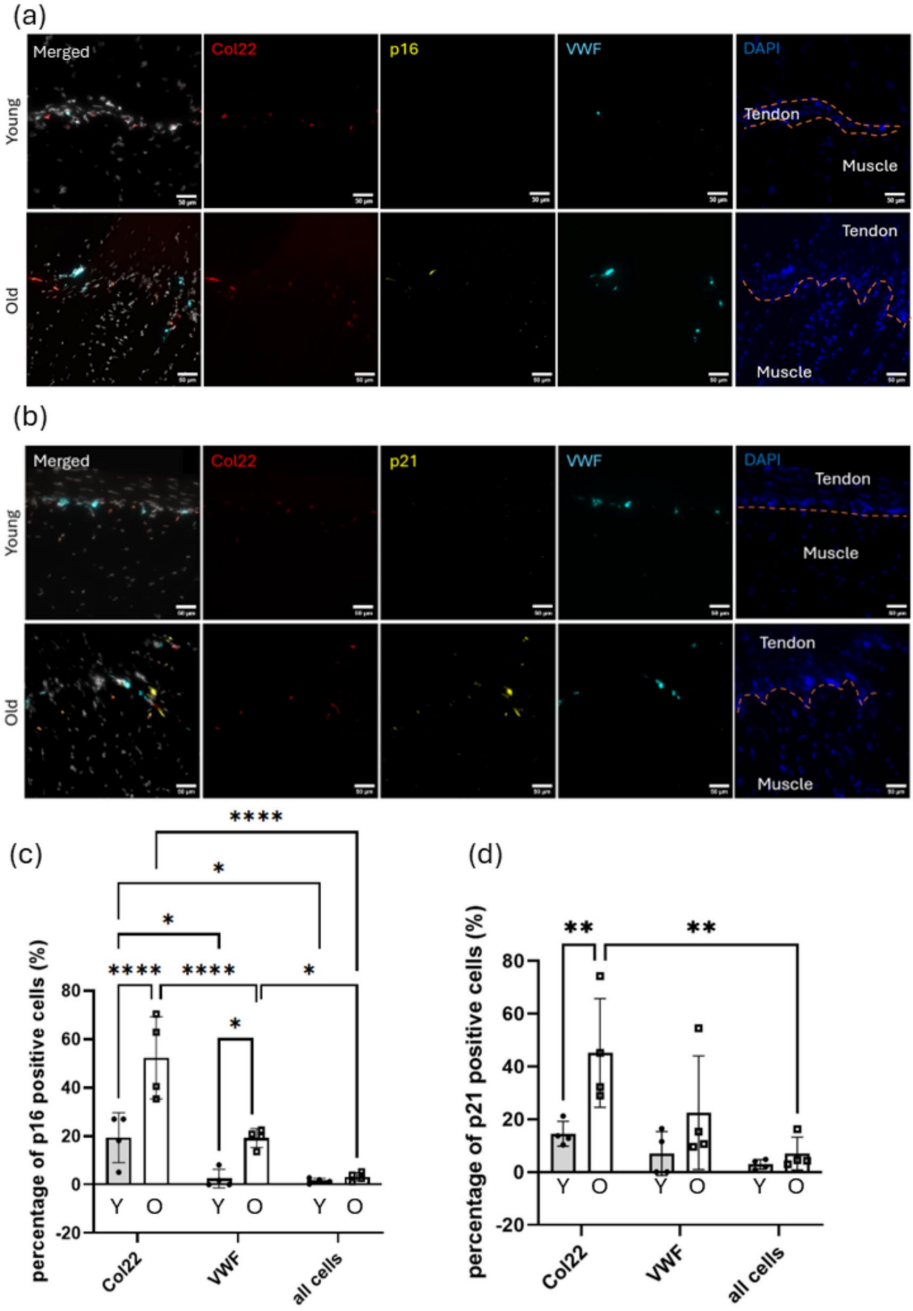
Senescence was detected more frequently in MTJ-specific and vascular endothelial cells compared to the overall cell population. Representative in situ hybridisation images of young and old mouse MTJ (n = 4 per age group) showing senescence markers (**a**) p16 and (**b**) p21.Red: Col22 (MTJ marker); yellow: (**a**) p16 and (**b**) p21; cyan: VWF (endothelial cell marker); grey: DAPI. Blue DAPI image on the right shows the interface between the muscle and tendon indicated by orange dashed lines. Scale bar is 50 µm. Percentage of (**c**) p16 and (**d**) p21 positive cells were measured in Col22-positive MTJ-specific cells, VWF-positive endothelial cells, and all cells in the field of view. Y: young; O: old. Two-way ANOVA followed by Tukey’s multiple comparisons test was used to calculate the significance between the age groups and cell populations. Data are presented as mean ± SD: *p < 0.05 **p < 0.01 ****p < 0.0001

In situ hybridisation, a technique used to visualise RNA expression within tissue sections, was employed to detect senescence markers at the MTJ. In old MTJs, senescence markers p16 and p21 were predominantly co-localised with the MTJ marker Col22 and endothelial cell marker VWF (Fig. 3a, b). Col22-positive MTJ-specific cells exhibited significantly higher percentages of p16- and p21-positive cells with age (270% and 310% increases, respectively), while VWF-positive endothelial cells showed a 780% increase in p16-positive cells with age (Fig. 3c, d).

While p16 expression was significantly higher in Col22-positive MTJ-specific cells (52%) and VWF-positive endothelial cells (19%) compared with all cells in the field of view (3.2%) in the old MTJ, p16 expression was also significantly higher in Col22-positive MTJ-specific cells (19%) than VWF-positive endothelial cells (2.5%) and all cells (1.6%) in the young MTJ. Similarly, p21 expression was significantly higher in Col22-positive MTJ-specific cells (45%) than in all cells in the old MTJ, whereas no significant difference was observed between VWF-positive endothelial cells and the overall cell population. However, in young MTJ, there was no significant difference in p21 expression among different cell types.

**Fig. 4:**
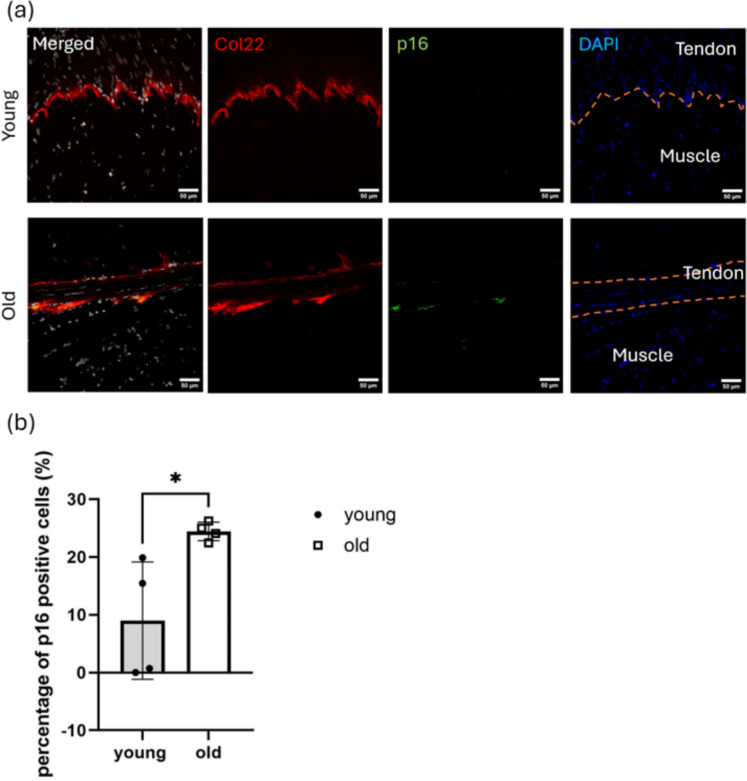
Protein expression analysis showed an increase in p16-positive cell proportion within Col22-positive MTJ-specific cells with age. **a** Representative immunofluorescent images of young and old MTJ (n = 4 per age group). Red: Col22; green: p16; grey: DAPI. Blue DAPI image on the right shows the interface between the muscle and tendon indicated by orange dashed lines. Scale bar is 50 µm. **b** Percentage of p16 positive cells in Col22-positive MTJ-specific cell population. Data are presented as mean ± SD (n = 4). A Mann-Whitney test was used to calculate the significance between young and old tendons. *p < 0.05

Expression of Col22 and p16 was assessed at the protein level using 2D immunofluorescent labelling. In old MTJ, expression of p16 was predominantly co-localised with Col22 expression (Fig. 4a), which was not observed in young MTJ. Quantitative analysis revealed a 270% increase in the proportion of p16-positive cells within the Col22-positive MTJ-specific cell population with age (Fig. 4b), consistent with the increase observed in the RNA expression analysis shown in Fig. 3.

## Discussion

This study provides the first comprehensive investigation of structural and cellular alterations with age in the mouse MTJ, demonstrating that age-related structural changes occur within the MTJ, which are accompanied by reduced vascularity and increased cell senescence in this region.

One of the limitations of this study was using mice immunised against the ancestral SARS-CoV-2 strain. However, both young and old mice were immunised simultaneously and underwent identical treatments after the immunisation. Although some individuals may experience musculoskeletal pain as a result of SARS-CoV-2 infection (Li et al. 2024), it has been reported that SARS-CoV-2 infection has no association with musculoskeletal function in humans (Reiter et al. 2025). In addition, the vaccine was administered into the right biceps femoris muscle, and the tissues obtained in this study were anatomically distinct from the injection site. Therefore, it is unlikely that the immunisation affected the MTJ structure, vasculature, or cellular senescence.

Another limitation of this study was the exclusive use of male mice. While restricting the cohort to males eliminated sex as a biological variable, it is also important to investigate sex-specific differences at the MTJ across ageing. Therefore, future studies should include both males and females to investigate potential sex-related differences in MTJ ageing.

Age-related structural changes in the MTJ have traditionally been investigated using 2D imaging approaches. In this study, µCT imaging was employed to explore structural changes in the MTJ with age in 3D, demonstrating a significant decrease in muscle fibre diameter. Similar findings have been reported by several previous studies using 2D imaging, with the reduction in size depending on the types of muscle fibres; type II muscle fibre size decreases more than type I muscle fibres with age (Lexell et al. 1988; Coggan et al. 1992; Frontera et al. 2000; Deschenes 2004; Nilwik et al. 2013; Callahan et al. 2014; Lee et al. 2024). In the current study, the mean muscle fibre diameter reduction with age was 27%, which is similar to the mean muscle fibre reduction reported previously in human quadriceps muscle (~ 20%) (Nilwik et al. 2013). In addition, pennation angle, which is the angle between the muscle fibres and tendon long axis, significantly decreased with age. A larger pennation angle results in increased force generation capacity in muscle (Lieber and Fridén 2000; Sopher et al. 2017) and therefore the decrease in pennation angle, combined with the reduction in muscle fibre diameter, strongly indicates a reduction in muscle force generation capacity in the MTJ region (Lieber and Fridén 2000; Krivickas et al. 2011; Sopher et al. 2017) and aligns with a previous report of age-related decreases in muscle force production observed in skeletal muscle (Zhang et al. 2020). Further analysis of µCT images showed a trend towards an age-related increase in MTJ surface area, which has also been reported in 2D image analysis of mouse soleus MTJ (Nielsen et al. 2018). The structural alterations observed with age in this study indicate that mouse Achilles MTJ may undergo functional deterioration and loss of force generation capacity with age. However, age-related alterations in MTJ mechanical properties and force generating capacity were not directly measured in the current study and therefore remain an important area for future investigation. In addition, it remains to be established whether the age-related changes observed in this study replicate those seen in the human MTJ.

Three-dimensional visualisation of muscle-tendon unit vascularity was achieved by confocal imaging of whole-tissue immunolabelled for VWF to target endothelial cell populations, and LAMA2 labelling for muscle cell populations. Imaging revealed the presence of vasculature within both muscle and tendon tissues, as well as across their interface, the MTJ. Quantitative analysis showed a significant reduction in VWF-labelled endothelial cell volume normalised by the whole tissue volume, but no significant difference in LAMA2-labelled muscle volume with age, indicating an age-associated decline in vascularisation at the muscle-tendon unit without a corresponding reduction in muscle volume, which supports the findings from the µCT image analysis. Together with previous reports of vascular decline in aged muscle and tendon (Brewer 1979; Márquez-Arabia et al. 2017; Fukada and Kajiya 2020), these findings indicated that reduced vascularisation is a shared feature of musculoskeletal ageing across tissues, which may contribute to functional deterioration at the MTJ through limited delivery of oxygen, amino acids, nutrients and hormones (Landers-Ramos and Prior 2018). For future studies, it would be interesting to directly compare age-related vascular alterations at the MTJ with those occurring in the adjacent muscle and tendon tissues.

In situ hybridisation was employed to investigate the expression of senescence markers, p16 and p21, in different cell populations in the MTJ. Both markers were predominantly localised to Col22-positive MTJ-specific cells and, to a lesser extent, VWF-positive endothelial cells. These results suggest that these cell types are particularly susceptible to senescence with age compared to the rest of the cell populations in the MTJ, with particularly pronounced vulnerability observed in Col22-positive MTJ-specific cells. Additionally, in Col22-positive MTJ-specific cells, elevated expression of p16 was observed in young MTJ compared to the other cell types, suggesting that Col22-positive MTJ-specific cells may experience senescence in early age. Further investigations are required to elucidate the mechanisms underlying the apparent susceptibility of Col22-positive MTJ-specific cells to senescence even in young MTJ.

The age-related accumulation of p16 in Col22-positive MTJ-specific cells was further validated at the protein level using immunolabelling. The marked increases in p16 and p21 expression within Col22-positive MTJ-specific cells indicated that MTJ-specific cells are prone to senescence, which may impair junctional integrity and contribute to age-related disorders and functional decline of the MTJ. Supporting this notion, the development of age-related pathologies and tissue dysfunction induced by upregulation of p16 and p21 has previously been observed in muscle (Baker et al. 2011; Englund et al. 2023). In addition, a study has shown that Col22 is a key factor maintaining skeletal muscle strength and force transmission capacity at the MTJ (Malbouyres et al. 2022), which may further support the potential functional deterioration and force generation capacity at the MTJ with age. Similarly, the elevated p16 expression observed in VWF-positive endothelial cells suggests induction of vascular cell senescence, which may result in vascular dysfunction, disrupted vascular ECM formation and fibrosis (Grosse et al. 2020; Graves et al. 2025), further exacerbating MTJ degeneration. However, the extent to which these mechanisms translate to human MTJ ageing remains to be determined.

Col22 was employed as an MTJ marker in this study. Although Col22 is a well-established and widely used marker of the myotendinous junction, several additional, less extensively characterised MTJ-associated markers, such as paxillin and talin, have been identified and utilised in previous studies (Tidball et al. 1986; Turner et al. 1991; Conti et al. 2009; Charvet et al. 2012). Future investigations could incorporate these markers to more comprehensively define MTJ-specific cell populations.

While senescence in Col22-positive MTJ-specific cells and VWF-positive endothelial cells was demonstrated in this study, and Col22 plays an important role in vascular integrity (Ton et al. 2018), it remains unclear whether these two cell types influence each other during ageing. Future studies should investigate the relationship between MTJ-specific cells and vascular endothelial cells in the context of ageing, potentially using in vitro MTJ co-culture models. Despite the high incidence of MTJ injuries, current treatments remain insufficient (Yan et al. 2018; Narayanan and Calve 2021). MTJ regeneration, and the effects of ageing, are poorly understood at cellular and ultrastructure level (Mackey 2024), and it is essential to understand the regeneration mechanisms in the MTJ to develop more effective treatments. The results in this study revealed that Col22-positive MTJ-specific cells undergo senescence earlier and more extensively than other cell types, suggesting that these cells could be a new promising therapeutic target to preserve MTJ integrity and function during ageing. Immunotherapies offer the potential for target-specific interventions and can be combined with senolytic agents that selectively eliminate senescent cells (Suda et al. 2025). Immunotherapies aimed at Col22-positive MTJ cells could therefore be explored as a novel approach to enhance MTJ health and structural integrity.

## Conclusion

This study provides the first comprehensive characterisation of age-related structural and cellular changes within the mouse MTJ. High-resolution µCT and confocal imaging revealed that ageing leads to a reduction in muscle fibre diameter and vascular volume at the muscle-tendon unit, alongside an expansion of the MTJ interface area, indicating altered force generation capacity and compromised tissue integrity. Cellular analysis demonstrated that both MTJ-specific Col22-positive cells and VWF-positive endothelial cells exhibit increased expression of senescence markers with age, suggesting that these cell types are particularly vulnerable to age-associated dysfunction. Together, these findings highlight that ageing drives structural remodelling and cell-type-specific senescence within the mouse MTJ, which may contribute to impaired tissue functionality and repair capacity. These findings provide a useful framework for understanding potential mechanisms of MTJ ageing, which may lead to the development of therapeutic approaches for preserving MTJ integrity and function in older individuals.

## Supplementary Information

Below is the link to the electronic supplementary material.

Supplementary file1 (DOCX 379 KB)

## Data Availability

The data that support the outcomes of this study are available from the paper and supporting material. Upon request, raw data are available from the corresponding author.

## References

[CR1] Baker DJ et al (2011) Clearance of p16 Ink4a-positive senescent cells delays ageing-associated disorders. Nature 479(7372):232–236. 10.1038/nature10600 22048312 10.1038/nature10600 PMC3468323

[CR2] Bayrak E, Yilgor Huri P (2018) Engineering musculoskeletal tissue interfaces. Front Mater 5(April):1–8. 10.3389/fmats.2018.00024

[CR3] Bloom SI, Islam MT, Lesniewski LA, Donato AJ (2023) Mechanisms and consequences of endothelial cell senescence. Nat Rev Cardiol 20(1):38–51. 10.1038/s41569-022-00739-0 35853997 10.1038/s41569-022-00739-0 PMC10026597

[CR4] Brewer BJ (1979) Aging of the rotator cuff. Am J Sports Med 7(2):102–110. 10.1177/036354657900700206 434288 10.1177/036354657900700206

[CR5] Callahan DM, Bedrin NG, Subramanian M, Berking J, Ades PA, Toth MJ, Miller MS (2014) Age-related structural alterations in human skeletal muscle fibers and mitochondria are sex specific: relationship to single-fiber function. J Appl Physiol 116(12):1582–1592 24790014 10.1152/japplphysiol.01362.2013 PMC4064376

[CR6] Charvet B, Ruggiero F, Le Guellec D (2012) The development of the myotendinous junction. A review. Muscles, Ligaments and Tendons Journal 2(2):53–63 23738275 PMC3666507

[CR7] Clarkson PM, Hubal MJ (2002) Exercise-induced muscle damage in humans. Am J Phys Med Rehabil 81(Supplement):S52–S69 12409811 10.1097/00002060-200211001-00007

[CR8] Coggan AR et al (1992) Histochemical and enzymatic comparison of the gastrocnemius muscle of young and elderly men and women. J Gerontol 47(3):B71–B76. 10.1093/geronj/47.3.B71 1573181 10.1093/geronj/47.3.b71

[CR9] Conti FJ, Monkley SJ, Wood MR, Critchley DR, Müller U (2009) Talin 1 and 2 are required for myoblast fusion, sarcomere assembly and the maintenance of myotendinous junctions. Development 136(21):3597–3606. 10.1242/dev.035857 19793892 10.1242/dev.035857 PMC2761109

[CR10] Čretnik A, Košir R (2023) Incidence of Achilles tendon rupture: 25-year regional analysis with a focus on bilateral ruptures. J Int Med Res. 10.1177/03000605231205179 37976267 10.1177/03000605231205179 PMC10657533

[CR11] Deschenes MR (2004) Effects of aging on muscle fibre type and size. Sports Med 34(12):809–824 15462613 10.2165/00007256-200434120-00002

[CR12] Dutta S, Sengupta P (2016) Men and mice: relating their ages. Life Sci 152:244–248. 10.1016/j.lfs.2015.10.025 26596563 10.1016/j.lfs.2015.10.025

[CR13] Englund DA et al (2023) p21 induces a senescence program and skeletal muscle dysfunction. Mol Metab 67:101652. 10.1016/j.molmet.2022.101652 36509362 10.1016/j.molmet.2022.101652 PMC9800630

[CR14] Frontera WR, Suh D, Krivickas LS, Hughes VA, Goldstein R, Roubenoff R (2000) Skeletal muscle fiber quality in older men and women. Am J Physiol Cell Physiol 279(3):C611–C618 10942711 10.1152/ajpcell.2000.279.3.C611

[CR15] Fukada K, Kajiya K (2020) Age-related structural alterations of skeletal muscles and associated capillaries. Angiogenesis 23(2):79–82. 10.1007/s10456-020-09705-1 31993832 10.1007/s10456-020-09705-1

[CR16] Garrett WE Jr, Faherty MS (2017) Muscle-tendon junction injury. Muscle and Tendon Injuries: Evaluation and Management. Springer, Berlin Heidelberg, pp 51–60. 10.1007/978-3-662-54184-5_5

[CR17] Grange S, Reurink G, Nguyen AQ, Riviera-Navarro C, Foschia C, Croisille P, Edouard P (2023) Location of hamstring injuries based on magnetic resonance imaging: a systematic review. Sports Health 15(1):111–123. 10.1177/19417381211071010 35148645 10.1177/19417381211071010 PMC9808837

[CR18] Graves SI, Meyer CF, Jeganathan KB, Baker DJ (2025) p16-expressing microglia and endothelial cells promote tauopathy and neurovascular abnormalities in PS19 mice. Neuron 113(14):2251-2264.e4. 10.1016/j.neuron.2025.04.020 40381614 10.1016/j.neuron.2025.04.020 PMC12289416

[CR19] Grosse L, Wagner N, Emelyanov A, Molina C, Lacas-Gervais S, Wagner K-D, Bulavin DV (2020) Defined p16High senescent cell types are indispensable for mouse healthspan. Cell Metab 32(1):87-99.e6. 10.1016/j.cmet.2020.05.002 32485135 10.1016/j.cmet.2020.05.002

[CR20] Han Y, Kim SY (2023) Endothelial senescence in vascular diseases: current understanding and future opportunities in senotherapeutics. Exp Mol Med 55(1):1–12. 10.1038/s12276-022-00906-w 36599934 10.1038/s12276-022-00906-w PMC9898542

[CR21] Han W, Wang B, Liu J, Chen L (2017) The p16/miR-217/EGR1 pathway modulates age-related tenogenic differentiation in tendon stem/progenitor cells. Acta Biochim Biophys Sin 49(11):1015–1021. 10.1093/abbs/gmx104 29036495 10.1093/abbs/gmx104

[CR22] Ho G, Tantigate D, Kirschenbaum J, Greisberg JK, Vosseller JT (2017) Increasing age in Achilles rupture patients over time. Injury 48(7):1701–1709. 10.1016/j.injury.2017.04.007 28457569 10.1016/j.injury.2017.04.007

[CR23] Huijing PA (1999) Muscle as a collagen fiber reinforced composite: a review of force transmission in muscle and whole limb. J Biomech 32(4):329–345. 10.1016/S0021-9290(98)00186-9 10213024 10.1016/s0021-9290(98)00186-9

[CR24] Iwasaki N, Karali A, Roldo M, Blunn G (2024a) Full-field strain measurements of the muscle-tendon junction using X-ray computed tomography and digital volume correlation. Bioengineering 11(2):162 38391648 10.3390/bioengineering11020162 PMC10886230

[CR25] Iwasaki N, Roldo M, Karali A, Sensini A, Blunn G (2024b) Development of muscle tendon junction in vitro using aligned Electrospun PCL fibres. Engineered Regeneration 5(3):409–420

[CR26] Iwasaki N, Morrison B, Karali A, Roldo M, Blunn G (2025) Measuring full-field strain of the muscle-tendon junction using confocal microscopy combined with digital volume correlation. J Mech Behav Biomed Mater 164:106925 39938281 10.1016/j.jmbbm.2025.106925

[CR27] Iwasaki N, Llewellyn J, Brown J, Zamboulis DE, Finding EJT, Wheeler‐Jones CPD, Thorpe CT (2026) Immunolabelling and micro‐computed tomography revealed age‐related alterations in 3D microvasculature of tendons. Aging Cell. 10.1111/acel.70293 41250917 10.1111/acel.70293 PMC12740099

[CR28] Koch M et al (2004) A novel marker of tissue junctions, collagen XXII. J Biol Chem 279(21):22514–22521 15016833 10.1074/jbc.M400536200 PMC2925840

[CR29] Krivickas LS, Dorer DJ, Ochala J, Frontera WR (2011) Relationship between force and size in human single muscle fibres. Exp Physiol 96(5):539–547. 10.1113/expphysiol.2010.055269 21317219 10.1113/expphysiol.2010.055269

[CR30] Landers-Ramos RQ, Prior SJ (2018) The microvasculature and skeletal muscle health in aging. Exerc Sport Sci Rev 46(3):172–179. 10.1249/JES.0000000000000151 29652695 10.1249/JES.0000000000000151 PMC6005745

[CR31] Lee C, Woods PC, Paluch AE, Miller MS (2024) Effects of age on human skeletal muscle: a systematic review and meta-analysis of myosin heavy chain isoform protein expression, fiber size, and distribution. Am J Physiol Cell Physiol 327(6):C1400–C1415. 10.1152/ajpcell.00347.2024 39374077 10.1152/ajpcell.00347.2024 PMC11684863

[CR32] Lexell J, Taylor CC, Sjöström M (1988) What is the cause of the ageing atrophy? J Neurol Sci 84(2–3):275–294. 10.1016/0022-510X(88)90132-3 3379447 10.1016/0022-510x(88)90132-3

[CR33] Li H et al (2024) The impact of COVID-19 infection on musculoskeletal pain and its associating factors: a cross-sectional study. Front Public Health. 10.3389/fpubh.2024.1422659 39257944 10.3389/fpubh.2024.1422659 PMC11384986

[CR34] Lieber RL, Fridén J (2000) Functional and clinical significance of skeletal muscle architecture. Muscle Nerve 23(11):1647–1666. 10.1002/1097-4598(200011)23:11<1647::AID-MUS1>3.0.CO;2-M 11054744 10.1002/1097-4598(200011)23:11<1647::aid-mus1>3.0.co;2-m

[CR35] Mackey AL (2024) The Myotendinous junction—form and function. Cold Spring Harb Perspect Biol. 10.1101/cshperspect.a041500 10.1101/cshperspect.a041500 PMC12401052 39134380

[CR36] Malbouyres M et al (2022) Lack of the myotendinous junction marker col22a1 results in posture and locomotion disabilities in zebrafish. Matrix Biol 109:1–18. 10.1016/j.matbio.2022.03.002 35278627 10.1016/j.matbio.2022.03.002

[CR37] Marqueti RC et al (2018) Effects of aging and resistance training in rat tendon remodeling. FASEB J 32(1):353–368. 10.1096/fj.201700543r 28899880 10.1096/fj.201700543R

[CR38] Márquez-Arabia WH et al (2017) Influence of aging on microvascular supply of the Gluteus Medius tendon: a cadaveric and histologic study. Arthroscopy 33(7):1354–1360. 10.1016/j.arthro.2017.01.036 28390662 10.1016/j.arthro.2017.01.036

[CR39] Marr N, Hopkinson M, Hibbert AP, Pitsillides AA, Thorpe CT (2020) Bimodal whole-mount imaging of tendon using confocal microscopy and X-ray micro-computed tomography. Biological Procedures Online. 10.1186/s12575-020-00126-4 32624710 10.1186/s12575-020-00126-4 PMC7329428

[CR40] McCarthy MM, Hannafin JA (2014) The mature athlete. Sports Health 6(1):41–48. 10.1177/1941738113485691 24427441 10.1177/1941738113485691 PMC3874221

[CR41] Møbjerg A et al (2025) Spatially distinct ECM-producing fibroblasts and myonuclei orchestrate early adaptation to mechanical loading in the human muscle-tendon unit. Am J Physiol-Cell Physiol. 10.1101/2025.08.28.672815 41117483 10.1152/ajpcell.00700.2025

[CR42] Nadon NL (2004) Maintaining aged rodents for biogerontology research. Lab Anim 33(8):36–41. 10.1038/laban0904-36 10.1038/laban0904-36 15334110

[CR43] Narayanan N, Calve S (2021) Extracellular matrix at the muscle–tendon interface: functional roles, techniques to explore and implications for regenerative medicine. Connect Tissue Res 62(1):53–71. 10.1080/03008207.2020.1814263 32856502 10.1080/03008207.2020.1814263 PMC7718290

[CR44] Nielsen KB, Lal NN, Sheard PW (2018) Age-related remodelling of the myotendinous junction in the mouse soleus muscle. Exp Gerontol 104:52–59 29421351 10.1016/j.exger.2018.01.021

[CR45] Nilwik R, Snijders T, Leenders M, Groen BBL, van Kranenburg J, Verdijk LB, Van Loon LJC (2013) The decline in skeletal muscle mass with aging is mainly attributed to a reduction in type II muscle fiber size. Exp Gerontol 48(5):492–498. 10.1016/j.exger.2013.02.012 23425621 10.1016/j.exger.2013.02.012

[CR46] Pedret C, Balius R, Blasi M, Dávila F, Aramendi JF, Masci L, de la Fuente J (2020) Ultrasound classification of medial gastrocnemious injuries. Scand J Med Sci Sports 30(12):2456–2465. 10.1111/sms.13812 32854168 10.1111/sms.13812

[CR47] Petrany MJ et al (2020) Single-nucleus RNA-seq identifies transcriptional heterogeneity in multinucleated skeletal myofibers. Nat Commun. 10.1038/s41467-020-20063-w 33311464 10.1038/s41467-020-20063-w PMC7733460

[CR48] Reiter A, Haack A, Petersen EL, Blankenberg S, Frosch KH, Thiesen D, Keller J (2025) Influence of the SARS-CoV-2 pandemic and infection on musculoskeletal function. Sci Rep. 10.1038/s41598-025-17780-x 40940387 10.1038/s41598-025-17780-x PMC12432240

[CR49] Rudzki JR et al (2008) Contrast-enhanced ultrasound characterization of the vascularity of the rotator cuff tendon: age- and activity-related changes in the intact asymptomatic rotator cuff. J Shoulder Elbow Surg 17(1):S96–S100 10.1016/j.jse.2007.07.004 18069013

[CR50] Saito Y, Yamamoto S, Chikenji TS (2024) Role of cellular senescence in inflammation and regeneration. Inflamm Regen. 10.1186/s41232-024-00342-5 38831382 10.1186/s41232-024-00342-5 PMC11145896

[CR51] Socha MJ, Segal SS (2018) Microvascular mechanisms limiting skeletal muscle blood flow with advancing age. J Appl Physiol (1985) 125(6):1851–1859. 10.1152/japplphysiol.00113.2018 30412030 10.1152/japplphysiol.00113.2018 PMC6737458

[CR52] Sopher RS, Amis AA, Davies DC, Jeffers JR (2017) The influence of muscle pennation angle and cross-sectional area on contact forces in the ankle joint. J Strain Anal Eng des 52(1):12–23. 10.1177/0309324716669250 29805194 10.1177/0309324716669250 PMC5952297

[CR53] Suda M, Tchkonia T, Kirkland JL, Minamino T (2025) Targeting senescent cells for the treatment of age-associated diseases. J Biochem 177(3):177–187. 10.1093/jb/mvae091 39727337 10.1093/jb/mvae091 PMC12987756

[CR54] Tidball JG (1983) The geometry of actin filament-membrane associations can modify adhesive strength of the myotendinous junction. Cell Motil 3(5):439–447 6607113 10.1002/cm.970030512

[CR55] Tidball JG, O’Halloran T, Burridge K (1986) Talin at myotendinous junctions. J Cell Biol 103(4):1465–1472. 10.1083/jcb.103.4.1465 3095335 10.1083/jcb.103.4.1465 PMC2114324

[CR56] Ton QV et al (2018) Collagen COL22A1 maintains vascular stability and mutations in COL22A1 are potentially associated with intracranial aneurysms. Dis Model Mech. 10.1242/dmm.033654 30541770 10.1242/dmm.033654 PMC6307901

[CR57] Turner CE, Kramarcy N, Sealock R, Burridge K (1991) Localization of paxillin, a focal adhesion protein, to smooth muscle dense plaques, and the myotendinous and neuromuscular junctions of skeletal muscle. Exp Cell Res 192(2):651–655. 10.1016/0014-4827(91)90090-H 1899076 10.1016/0014-4827(91)90090-h

[CR58] Vanhooren V, Libert C (2013) The mouse as a model organism in aging research: usefulness, pitfalls and possibilities. Ageing Res Rev 12(1):8–21. 10.1016/j.arr.2012.03.010 22543101 10.1016/j.arr.2012.03.010

[CR59] Vila Pouca MCP, Parente MPL, Jorge RMN, Ashton-Miller JA (2021) Injuries in muscle-tendon-bone units: a systematic review considering the role of passive tissue fatigue. Orthop J Sports Med 9(8):1–15. 10.1177/23259671211020731 10.1177/23259671211020731 PMC8361535 34395681

[CR60] Yan Z, Yin H, Nerlich M, Pfeifer CG, Docheva D (2018) Boosting tendon repair: interplay of cells, growth factors and scaffold-free and gel-based carriers. J Exp Orthop 5(1):1–13. 10.1186/s40634-017-0117-1 29330711 10.1186/s40634-017-0117-1 PMC5768579

[CR61] Yaseen W, Kraft-Sheleg O, Zaffryar-Eilot S, Melamed S, Sun C, Millay DP, Hasson P (2021) Fibroblast fusion to the muscle fiber regulates myotendinous junction formation. Nat Commun. 10.1038/s41467-021-24159-9 34158500 10.1038/s41467-021-24159-9 PMC8219707

[CR62] Zamboulis DE, Marr N, Lenzi L, Birch HL, Screen HRC, Clegg PD, Thorpe CT (2024) The interfascicular matrix of energy storing tendons houses heterogenous cell populations disproportionately affected by aging. Aging Dis 15(1):295 37307816 10.14336/AD.2023.0425-1 PMC10796100

[CR63] Zhang Y et al (2020) Microstructural analysis of skeletal muscle force generation during aging. Int J Numer Methods Biomed Eng. 10.1002/cnm.3295 10.1002/cnm.3295 PMC8080883 31820588

[CR64] Zhao C et al (2018) Preparation of decellularized biphasic hierarchical myotendinous junction extracellular matrix for muscle regeneration. Acta Biomater 68:15–28. 10.1016/j.actbio.2017.12.035 29294376 10.1016/j.actbio.2017.12.035

