# Supplementary material for: Ageing-related structural and cellular alterations in the mouse muscle-tendon junction
Source: Biogerontology. 2026 Apr 4;27(3):80. doi: 10.1007/s10522-026-10428-x (PMC13050345; doi:10.1007/s10522-026-10428-x)
Supplement: Supplementary file 1 — Supplementary file1 (DOCX 379 KB) [file 10522_2026_10428_MOESM1_ESM.docx]

**Supplementary data**

**
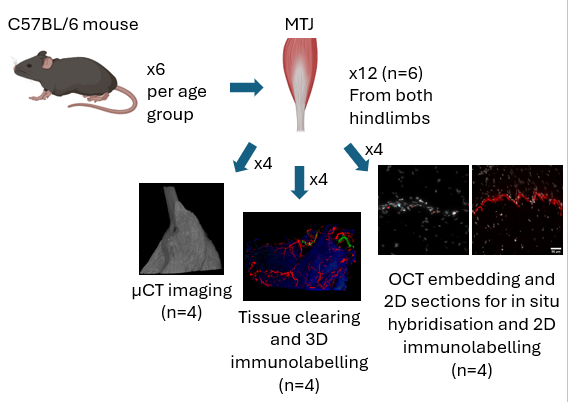
**

*Figure S1. Overview of sample allocation for each method.*


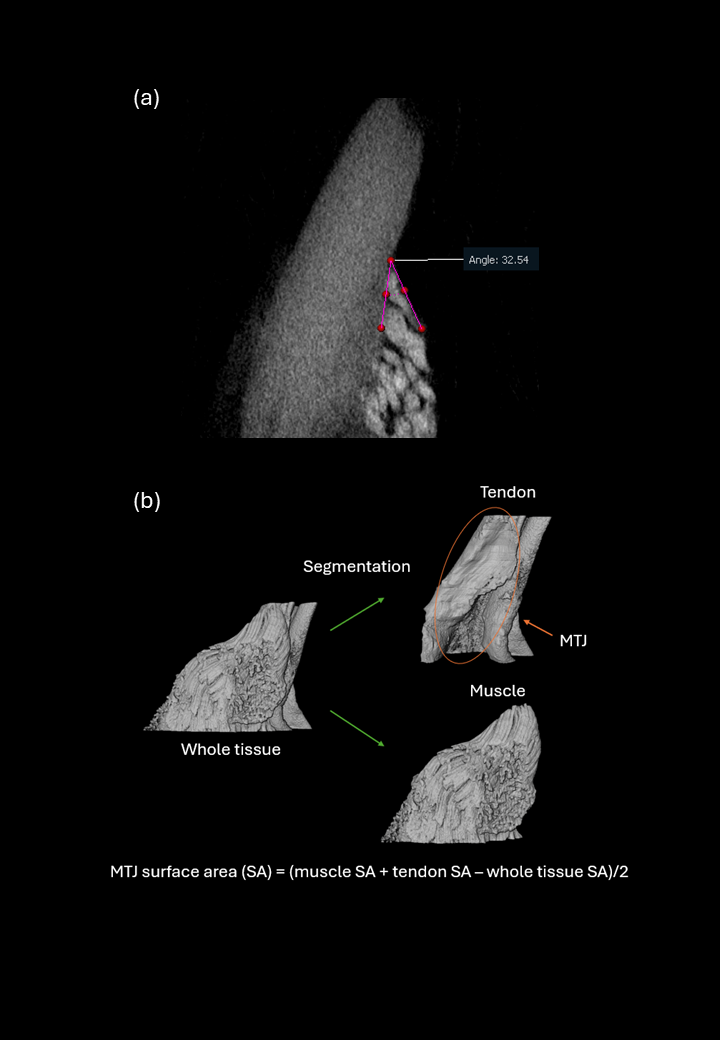


*Figure S2. Representative reconstructed µCT images of young muscle-tendon unit illustrating (a) pennation angle measurement from a 2D slice and (b) the segmentation and MTJ surface area measurements. Pennation angle measurement was performed for all identifiable interfaces both medially and laterally. Surface area of each tissue was measured using volume fraction function in Avizo.*

*
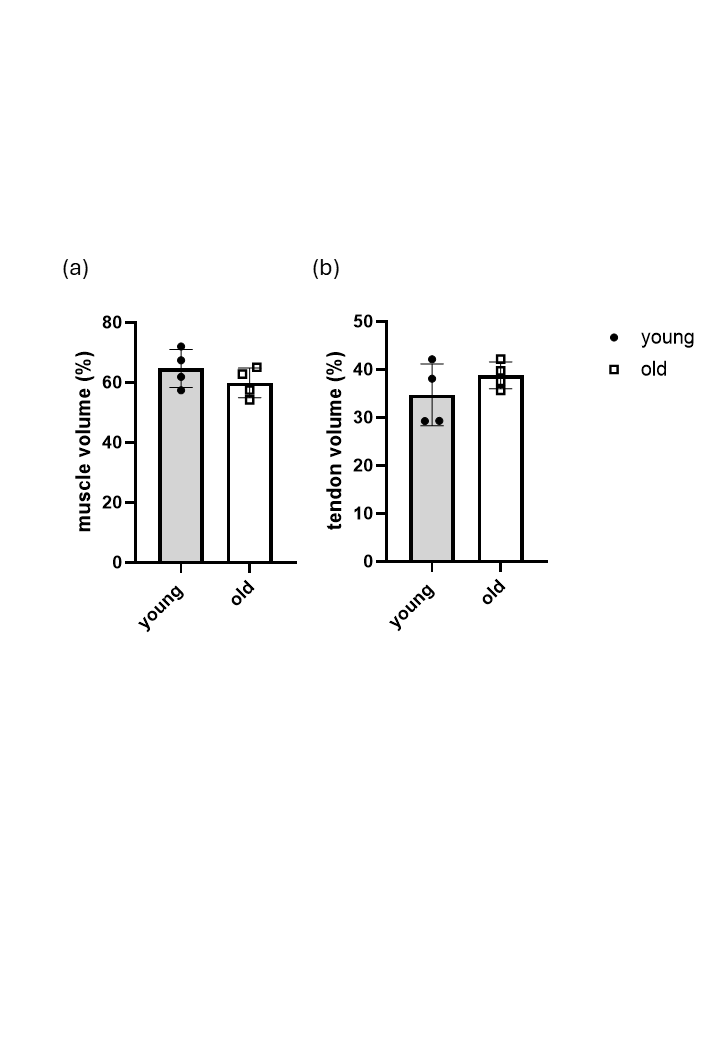
*

*Figure S3. Quantitative analysis of µCT images showing (a) muscle volume and (b) tendon volume at the MTJ normalised by the whole MTJ volume. Data are presented as mean ± SD from analysis of 4 young and 4 old mice. Mann-Whitney test was used to calculate the significance between young and old MTJs, and no significant difference was detected.*
